# Supplementary material for: Atomic Layer Deposition of Cobalt Catalyst for Fischer–Tropsch Synthesis in Silicon Microchannel Microreactor
Source: Nanomaterials (Basel). 2022 Jul 15;12(14):2425. doi: 10.3390/nano12142425 (PMC9320865; doi:10.3390/nano12142425)
Supplement: Supplementary file 1 [file nanomaterials-12-02425-s001.zip › nanomaterials-1688332-supplementary.pdf]

nanomaterials

MDPI

Supplementary Materials

Atomic Layer Deposition of Cobalt Catalyst for Fischer–Tropsch Synthesis in Silicon Microchannel Microreactor

Nafeezuddin Mohammad [1], Shyam Aravamudhan [1] and Debasish Kuila [1,2,*]

[1] Department of Nanoengineering, Joint School of Nanoscience and Nanoengineering, Greensboro, NC 27401, USA; nmohammad@aggies.ncat.edu (N.M.); saravamu@ncat.edu (S.A.)
[2] Department of Chemistry, North Carolina A&T State University, Greensboro, NC 27411, USA
* Correspondence: dkuila@ncat.edu

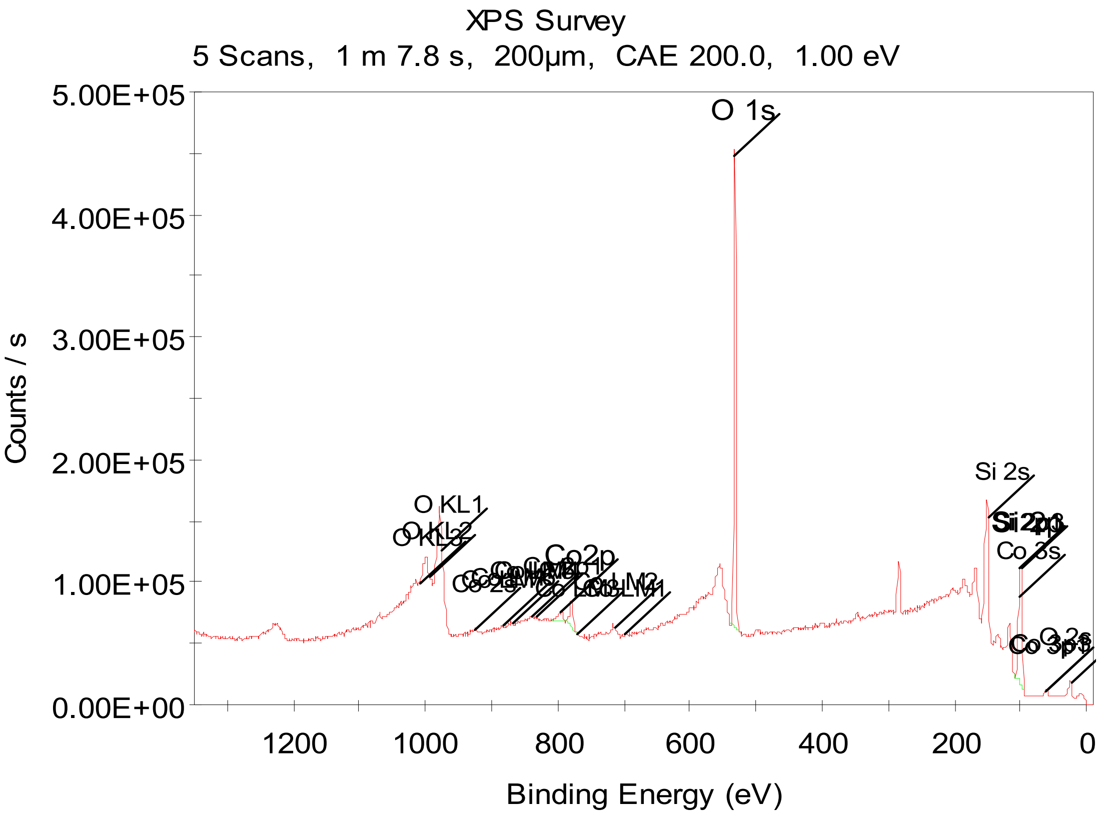

Figure S1. XPS survey of the fresh sample with no carbon peak.
